# Influence of triglycerides on the link between gamma-glutamyl transferase to high-density lipoprotein cholesterol ratio and nonalcoholic fatty liver disease in nonobese Chinese adults: A secondary cohort study

**DOI:** 10.1097/MD.0000000000042078

**Published:** 2025-04-11

**Authors:** Aihua Chen, Cishuang Fu, Haiying Chen

**Affiliations:** a Department of General Practice, First People’s Hospital of Changde City, Changde, Hunan, PR China.

**Keywords:** gamma-glutamyl transferase, high-density lipoprotein cholesterol, interaction, modification, nonalcoholic fatty liver disease, triglyceride

## Abstract

Nonalcoholic fatty liver disease (NAFLD) represents a chronic hepatic disorder marked by excessive lipid deposition within liver tissues. Previous studies have identified the gamma-glutamyl transferase to high-density lipoprotein cholesterol (GGT/HDL-c) ratio as a potential marker associated with NAFLD risk. However, the influence of triglyceride (TG) levels on this association has not been fully elucidated. This study was designed to assess whether TG modulates the relationship between GGT/HDL-c and the incidence of NAFLD, and to explore the possible interaction between TG and GGT/HDL-c in relation to NAFLD risk. This investigation was a secondary analysis based on data from a cohort study, encompassing 11,692 nonobese Chinese adults. Participants were stratified into 2 groups according to their triglyceride levels: hypertriglyceridemia (TG ≥ 1.7 mmol/L) and non-hypertriglyceridemia (TG < 1.7 mmol/L). Additionally, the GGT/HDL-c ratio was divided into quartiles for detailed analysis. The association between GGT/HDL-c, TG, and the occurrence of NAFLD was evaluated using Cox proportional hazards regression models. Furthermore, the potential modifying effect of TG on the GGT/HDL-c–NAFLD relationship was examined by categorizing participants into 8 subgroups based on TG status and quartiles of GGT/HDL-c ratio. During a mean follow-up duration of 29.24 months, 1926 individuals (16.47%) were newly diagnosed with NAFLD. Both elevated GGT/HDL-c ratios and higher TG concentrations were significantly correlated with an increased risk of developing NAFLD. TG levels influenced the strength of the association between GGT/HDL-c and NAFLD. Specifically, the association was stronger among individuals without hypertriglyceridemia (HR = 1.019, 95% CI = 1.015–1.023, *P* < .0001), and comparatively weaker in the hypertriglyceridemia group (HR = 1.012, 95% CI = 1.008–1.016, *P* < .0001). Moreover, a significant interaction effect was observed between TG and GGT/HDL-c, with the greatest risk identified in participants exhibiting both elevated GGT/HDL-c ratios and high TG levels (HR = 6.662, 95% CI = 5.237–8.474, *P* < .0001). Triglyceride levels appear to modify the relationship between GGT/HDL-c and NAFLD risk, with a notable interaction effect between these 2 factors. Among nonobese Chinese adults, simultaneous management aimed at reducing both GGT/HDL-c ratios and TG concentrations may contribute to lowering the risk of NAFLD onset.

## 1. Introduction

Nonalcoholic fatty liver disease (NAFLD) is a chronic liver disease characterized by excessive fat accumulation in the liver (hepatic steatosis > 5% of hepatocytes) without significant alcohol consumption (<20 g/d for women, <30 g/d for men) or other secondary causes (e.g., viral hepatitis, steatogenic medications). NAFLD is a chronic liver disease characterized by excessive fat accumulation in the liver.^[1]^ NAFLD is commonly considered a manifestation of metabolic syndrome, often accompanied by type 2 diabetes, hypertension, dyslipidemia, and cardiovascular disease.^[2]^ The worldwide occurrence of NAFLD is estimated to be approximately 25%, and it is continuing to rise with the increasing prevalence of obesity and type 2 diabetes.^[3,4]^ NAFLD is associated with various severe complications, including cirrhosis, hepatocellular carcinoma, and increased liver-related mortality.^[3,5]^ Additionally, NAFLD is associated with a higher risk of cardiovascular disease, chronic kidney disease, and various other systemic illnesses.^[6,7]^ Consequently, NAFLD not only poses a significant threat to individual health but also imposes a substantial economic burden on public health systems.^[3]^

Obesity is an important risk factor for NAFLD. Research from Singapore^[8]^ indicated that overweight individuals face a notably higher risk of developing NAFLD, with the risk being even greater for those who are obese. Moreover, the study also revealed that even after weight reduction, individuals with a previous history of obesity still have a higher risk of NAFLD. Additionally, NAFLD is not uncommon in the nonobese population. A meta-analysis revealed that the prevalence of NAFLD in nonobese individuals is 10.2%,^[9]^ and another study showed a NAFLD prevalence of 13.0%.^[10]^ Therefore, it is crucial to pay attention to NAFLD in the nonobese population as well.

Multiple studies have demonstrated that metabolic abnormalities, such as elevated levels of blood pressure, blood glucose, uric acid (UA), total cholesterol (TC), and triglycerides (TG), as well as low levels of high-density lipoprotein (HDL-c), are independent risk factors for NAFLD in nonobese populations.^[10,11]^ High triglyceride levels are particularly regarded as a key predictor of NAFLD in nonobese individuals.^[10,12]^ Additionally gamma-glutamyl transferase (GGT) has been found to be highly correlated with the incidence of NAFLD,^[13,14]^ and GGT levels have shown a positive correlation with the extent of liver fibrosis in patients with NAFLD.^[15]^ A cross-sectional study further explored the association between GGT/HDL-c ratio and NAFLD, and found that the prevalence of NAFLD increased by 0.3% with every 1-unit increase in the GGT/HDL-C ratio, and the AUC of the GGT/HDL-c ratio was significantly higher than that of GGT or HDL-C alone.^[16]^ Since both TG and the GGT/HDL-c ratio are associated with NAFLD, it remains unclear whether different TG levels may influence the relationship between the GGT/HDL-c ratio and NAFLD. This study seeks to assess if triglycerides serve as a modifying factor in the association between the GGT/HDL-c ratio and nonalcoholic fatty liver disease (NAFLD) risk. Furthermore, it explores the potential interaction between TG levels and the GGT/HDL-c ratio in relation to NAFLD occurrence.

## 2. Methods

### 2.1. Data source

This study was approved by the Ethics Committee of First People’s Hospital of Changde City. This study is a secondary analysis based on a cohort study. The data used in this study was obtained from the DATADRYAD database (https://datadryad.org/stash) provided by Sun et al from: Association of low-density lipoprotein cholesterol within the normal range and NAFLD in the nonobese Chinese population: a cross-sectional and longitudinal study. Dryad, Dataset, https://doi.org/10.5061/dryad.1n6c4.^[17]^

**Figure 1.**
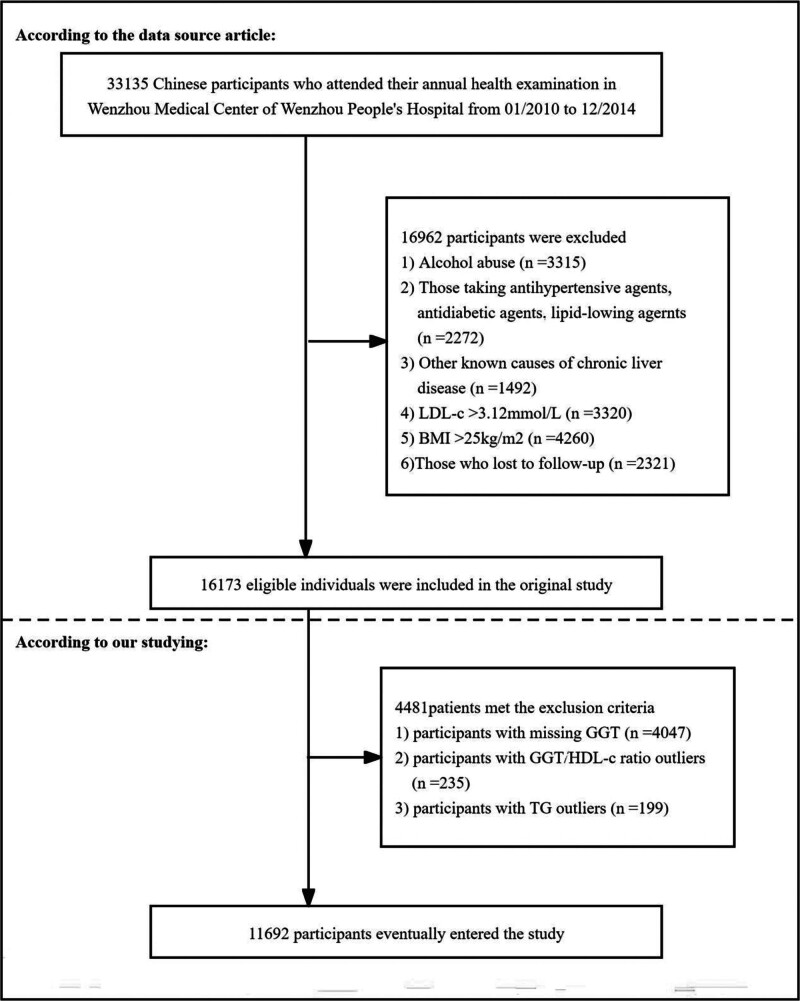
Flowchart of study participants.

The study population was recruited from healthy examines at the People’s Hospital of Wenzhou from January 2010 to December 2014. The original study included 33,135 nonobese participants without NAFLD. The study excluded the following participants: those with excessive alcohol consumption (>140 g/wk for men, >70 g/wk for women); those taking antihypertensive, antidiabetic, or lipid-lowering medications; those with a history of viral hepatitis, autoimmune hepatitis, or other known chronic liver diseases; those with body mass index (BMI) ≥ 25 kg/m²; those with LDL-c > 3.12 mmol/L; those lost to follow-up or with missing data. Finally, 16,173 nonobese participants without NAFLD were included in the original study. The study excluded individuals with incomplete GGT data and those whose TG or GGT/HDL-c ratio values were statistical outliers, identified as being beyond 3 standard deviations above or below the mean. This resulted in a final analytical sample of 11,692 participants (Fig. 1).^[17]^ Each participant voluntarily agreed to participate by signing an informed consent form. As this research is a secondary data analysis, the study does not require additional ethical approval.

### 2.2. Gamma-glutamyl transferase to high-density lipoprotein cholesterol ratio

GGT and HDL-c levels were measured by standard methods via an automated analyzer (Abbott AxSYM). The GGT/ HDL-c ratio is expressed as a continuous variable. GGT divided by HDL-c is the formula used to calculate the GGT/ HDL-C ratio, and the GGT and HDL-C levels were measured using a standard method with an automated analyzer (Abbott AxSYM). The GGT/HDL-C ratio was expressed as a continuous variable.

### 2.3. Triglyceride

TG were measured using an automated analyzer. Hypertriglyceridemia is characterized by serum TG levels ≥ 1.7 mmol/L, while non-hypertriglyceridemia is defined by TG levels < 1.7 mmol/L.^[18]^

### 2.4. Diagnosis of nonalcoholic fatty liver disease by ultrasonography

Nonalcoholic fatty liver disease was diagnosed in accordance with the guidelines established by the Chinese Association for the Study of Liver Disease.^[19]^ The diagnostic criteria for NAFLD included the presence of diffuse enhancement of near-field echoes in the liver region – greater than those observed in the kidney and spleen regions – along with progressive attenuation of far-field echoes. Additionally, diagnosis required the presence of at least 1 of the following sonographic features: obscured visualization of the intrahepatic lacunar structure; mild to moderate hepatomegaly characterized by a rounded and blunt liver margin; decreased hepatic blood flow signals, although the overall distribution of blood flow remained normal; or an indistinct or discontinuous appearance of the envelope of the right hepatic lobe and the diaphragm.

Participants underwent annual follow-up assessments over a 5-year observation period. The follow-up procedures were consistent with those conducted at baseline, including standardized hepatic ultrasonography performed in a blinded fashion to assess the development of NAFLD.

### 2.5. Other variables

As described in previous studies,^[17,20,21]^ general information, health habits, and medical history of the participants were collected by trained medical staff through standardized electronic forms. The BMI (kg/m^2^) was calculated as weight (kg) divided by height squared (m^2^). Blood pressure was taken in a calm setting with the participant seated, using an automatic blood pressure monitor. All biochemical parameters, such as alanine aminotransferase (ALT), aspartate aminotransferase (AST), alkaline phosphatase (ALP), albumin (ALB), globulin (GLB), total bilirubin (TBIL), direct bilirubin (DBIL), blood urea nitrogen (BUN), creatinine (Cr), UA, fasting plasma glucose (FPG), triglycerides (TG), TC, low-density lipoprotein cholesterol (LDL-c), HDL-c, and GGT, were assessed using standard procedures on an Abbott AxSYM automated analyzer.

### 2.6. Missing data processing

In observational studies, missing data is inevitable. In this study, the percentage of missing values for the variables was as follows: SBP and diastolic blood pressure (DBP) (0.13%), ALB and GLB (11.33%), TBIL (26.03%), DBIL (37.64%), BUN, Scr, UA, and FPG (all <0.01%). To address the potential biases caused by these missing data, we conducted multiple imputation analyses, using imputation models that included all the aforementioned variables.

### 2.7. Statistical analysis

Participants were stratified into 4 groups based on the quartile distribution of the GGT/HDL-c ratio. Continuous variables were expressed as mean ± standard deviation (SD) for normally distributed data or median (interquartile range) for non-normally distributed data. Categorical variables were presented as numbers (percentages). Group differences were assessed using ANOVA for normally distributed variables, the Kruskal–Wallis *H* test for skewed distributions, and the χ² test for categorical variables.

To examine the relationships between GGT/HDL-c, TG, and NAFLD risk, the following analyses were conducted: univariate and multivariate cox regression models 3 models were constructed:

### 2.8. Crude model: no adjustments

Model I: adjusted for sex, age, systolic blood pressure (SBP), DBP, and BMI.

Model II: further adjusted for additional covariates, including ALT, AST, ALB, GLB, ALP, TBIL, UA, FPG, serum creatinine (Scr), and LDL-c. Covariates were selected based on clinical relevance and prior research.^[22–24]^

Linear regression models with the same adjustment strategies were also used to explore the correlation between the GGT/HDL-c ratio and TG levels.

Participants were divided into hypertriglyceridemia (TG ≥ 1.7 mmol/L) and non-hypertriglyceridemia (TG < 1.7 mmol/L) groups. The association between GGT/HDL-c and NAFLD risk was evaluated separately within these groups using Cox proportional hazards regression models. An interaction test was performed to assess whether TG levels modified the relationship between GGT/HDL-c and NAFLD risk. A *P* value < 0.05 in the interaction test indicated a potential modifying effect. Smooth curve fitting was additionally employed to visualize the relationship between GGT/HDL-c and NAFLD risk across different TG levels.

Participants were further categorized into 8 groups based on GGT/HDL-c quartiles and the presence of hypertriglyceridemia. The reference group comprised individuals with non-hypertriglyceridemia and a GGT/HDL-c ratio < 10.68 (Q1). Cox proportional hazards regression models were used to examine the association between the remaining 7 groups and NAFLD risk. The interaction between TG and GGT/HDL-c was assessed by comparing effect sizes across groups.

All analyses were performed using EmpowerStats (https://www.empowerstats.com, X&Y Solutions, Inc., Boston) and R software (https://www.R-project.org, The R Foundation, R.4.2.0). A *P* value < .05 was considered statistically significant.

## 3. Results

### 3.1. Population characteristics

Baseline clinical and demographic characteristics by TG and GGT/HDL-C stratification of participants are shown in Table 1. The average age of the participants was 43.29 ± 14.97 years, and 6379 were male (54.56%). The median (Q1–Q3) of GGT/HDL-c ratio was 15.385 (10.674–23.358) and the mean ± SD of TG was 1.278 ± 0.61 mmol/L. We divided all participants into high triglyceride group (TG ≥ 1.7 mmol/L) and non-high triglyceride group (TG < 1.7 mmol/L), then each group was divided into 4 subgroups using the quartile of the GGT/HDL-c ratio. In groups with TG < 1.7 mmol/l, compared with the group Q1, the group Q4 was more male, older age, higher levels of BMI, SBP, DBP, ALT, AST, ALP, GLB, DBIL, BUN, Scr, UA, FPG, TG, LDL-c, GGT, and lower levels of HDL-c. Among groups with TG ≥ 1.7 mmol/l, group Q4 showed higher levels of BMI, SBP, DBP, ALT, AST, ALP, TBIL, Scr, UA, FPG, TG, and GGT, and lower levels of HDL-c compared to group Q1.

**Table 1:** The baseline characteristics of participants.

GGT/HDL-c quartile	TG < 1.7mmol/L				*P*	TG ≥ 1.7 mmol/L				*P*
	Q1 (<10.68)	Q2 (10.68–15.39)	Q3 (15.39–23.36)	Q4 (≥23.36)		Q1 (<10.68)	Q2 (10.68–15.39)	Q3 (15.39–23.36)	Q4 (≥23.36)	
N	2840	2641	2295	1628		81	267	644	1296	
Male	1403 (49.40%)	1415 (53.58%)	1301 (56.69%)	936 (57.49%)	<.001	43 (53.09%)	153 (57.30%)	359 (55.75%)	769 (59.34%)	.372
Age (yr)	42.55 ± 14.91	43.02 ± 14.91	43.42 ± 15.06	43.92 ± 14.76	.019	39.69 ± 14.92	44.73 ± 15.51	43.14 ± 15.45	44.49 ± 14.87	.014
BMI (kg/m^2^)	20.56 ± 1.94	21.14 ± 2.00	21.80 ± 1.98	22.27 ± 1.86	<.001	21.53 ± 1.85	22.01 ± 1.83	22.36 ± 1.66	22.89 ± 1.51	<.001
SBP (mm Hg)	115.33 ± 15.76	120.10 ± 16.44	124.11 ± 16.21	126.99 ± 16.62	<.001	122.78 ± 17.74	126.33 ± 16.77	126.49 ± 16.73	127.94 ± 15.82	.014
DBP (mm Hg)	69.56 ± 9.31	72.51 ± 9.98	74.30 ± 9.84	76.13 ± 10.22	<.001	73.41 ± 11.19	75.42 ± 10.36	76.20 ± 9.99	78.36 ± 10.36	<.001
ALT (U/L)	13.00 (10.00–16.00)	15.00 (11.00–19.00)	17.00 (13.00–23.00)	22.00 (17.00–30.25)	<.001	14.00 (11.00–17.00)	15.00 (12.00–19.00)	17.00 (13.00–22.00)	23.00 (18.00–31.00)	<.001
AST (U/L)	19.00 (17.00–22.00)	20.00 (18.00–24.00)	22.00 (19.00–25.00)	24.00 (21.00–29.00)	<.001	19.00 (18.00–22.00)	21.00 (19.00–25.00)	21.00 (18.00–25.00)	24.00 (21.00–28.00)	<.001
ALP (U/L)	62.91 ± 18.22	68.94 ± 19.50	73.66 ± 21.35	80.85 ± 29.33	<.001	70.31 ± 19.53	73.48 ± 19.67	77.21 ± 19.27	78.54 ± 20.14	<.001
ALB (g/L)	44.24 ± 2.60	44.52 ± 2.61	44.62 ± 2.65	44.37 ± 2.76	<.001	44.64 ± 2.74	44.61 ± 2.71	44.96 ± 2.61	44.99 ± 2.39	.107
GLB (g/L)	29.05 ± 3.69	29.30 ± 3.71	29.39 ± 3.68	29.67 ± 4.14	<.001	29.48 ± 3.65	29.56 ± 3.60	29.26 ± 3.59	29.24 ± 3.61	.588
TBIL (µmol/L)	11.81 ± 4.16	12.42 ± 4.27	13.00 ± 4.63	12.98 ± 5.21	<.001	11.88 ± 4.06	12.12 ± 4.66	12.72 ± 4.18	12.87 ± 4.44)	.023
DBIL (µmol/L)	2.08 (1.60–2.60)	2.20 (1.70–2.72)	2.29 (1.70–2.81)	2.31 (1.79–2.89)	<.001	2.00 (1.20–2.60)	2.00 (1.40–2.65	2.21 (1.60–2.78)	2.16 (1.60–2.71)	.074
BUN (µmol/L)	4.31 ± 1.33	4.55 ± 1.37	4.73 ± 1.29	4.89 ± 1.71	<.001	4.61 ± 1.25	4.70 ± 1.69	4.55 ± 1.37	4.62 ± 1.36	.498
Scr (mmol/L)	71.00 (63.00–81.00)	78.00 (67.00–93.00)	87.00 (75.00–97.00)	88.00 (77.00–97.25)	<.001	75.00 (66.00–88.00)	82.00 (70.00–95.50)	87.00 (76.00–97.00)	90.00 (79.00–98.00)	<.001
UA (µmol/L)	232.94 ± 73.91	274.17 ± 79.77	304.97 ± 77.40	326.43 ± 79.65	<.001	283.83 ± 85.65	305.99 ± 87.85	333.64 ± 82.14	352.37 ± 77.74	<.001
FPG (mmol/L)	5.04 ± 0.58	5.13 ± 0.73	5.22 ± 0.81	5.38 ± 1.05	<.001	4.97 ± 0.51	5.22 ± 0.55	5.26 ± 0.77	5.37 ± 0.94	<.001
TG (mmol/L)	0.88 ± 0.26	1.01 ± 0.2	1.13 ± 0.29	1.21 ± 0.29	<.001	2.04 ± 0.34	2.09 ± 0.40	2.19 ± 0.46	2.38 ± 0.54	<.001
TC (mmol/L)	4.61 ± 0.71	4.49 ± 0.72	4.47 ± 0.71	4.50 ± 0.70	<.001	5.18 ± 0.72	4.94 ± 0.66	4.84 ± 0.67	4.83 ± 0.68	<.001
LDL-c (mmol/L)	2.16 ± 0.47	2.23 ± 0.46	2.29 ± 0.47	2.30 ± 0.46	<.001	2.44 ± 0.44	2.40 ± 0.47	2.43 ± 0.43	2.42 ± 0.45	.800
HDL-c (mmol/L)	1.76 ± 0.31	1.51 ± 0.28	1.36 ± 0.29	1.33 ± 0.32	<.001	1.61 ± 0.28	1.38 ± 0.27	1.23 ± 0.25	1.15 ± 0.26	<.001
GGT (U/L)	14.00 (13.00–16.00)	19.00 (17.00–21.00)	24.00 (21.00–28.00)	42.00 (33.00–57.00)	<.001	14.00 (13.00–16.00)	18.00 (16.00–21.00)	23.00 (20.00–26.25)	40.00 (31.00–57.00)	<.001
GGT/HDL-c ratio	8.60 (7.39–9.64)	12.74 (11.68–13.99)	18.38 (16.67–20.46)	31.03 (26.26–41.51)	<.001	9.14 (8.09–10.06)	13.42 (12.07–14.62)	19.26 (17.30–21.13)	35.32 (28.57–48.98)	<.001

Values are n (%) or mean ± SD or median (quartile).

ALB = albumin, ALP = alkaline phosphatase, ALT = alanine aminotransferase, AST = aspartate aminotransferase, BMI = body mass index, BUN = serum urea nitrogen, DBIL = direct bilirubin, DBP = diastolic blood pressure, FPG = fasting plasma glucose, GGT = gamma-glutamyl transferase, GGT/HDL-c ratio = gamma-glutamyl transferase to high-density lipoprotein cholesterol ratio, GLB = globulin, HDL-C = high-density lipoprotein cholesterol, LDL-C = low-density lipid cholesterol, SBP = systolic blood pressure, Scr = serum creatinine, TBIL = total bilirubin, TC = total bcholesterol, TG = triglyceride, UA = uric acid.

### 3.2. The incidence rate of NAFLD

As shown in Table 2, over an average follow-up period of approximately 2.44 years, 1926 individuals (16.47%) developed nonalcoholic fatty liver disease (NAFLD). The cumulative incidence rate was 67.37 cases per 1000 person-years among all participants.In subgroups with TG levels < 1.7 mmol/L, the incidence of NAFLD varied across the 4 GGT/HDL-c ratio categories. Specifically, the lowest GGT/HDL-c ratio group had an NAFLD incidence of 2.99% (95% CI = 2.41%–3.67%), 8.41% (7.39%–9.51%), 15.82% (14.37%–17.35%), and 25.55% (23.48%–27.71%) respectively, and the cumulative incidence rate was 12.82, 34.36, 64.08, and 101.95 per 1000 person-years respectively. In groups with TG ≥ 1.7 mmol/l, the NAFLD incidence was 9.88% (4.69%–17.89%), 17.60% (13.38%–22.52%), 28.73% (25.33%–32.31%), and 46.30% (43.69%–49.02%) respectively, and The cumulative incidence rate was 37.11, 66.63, 110.36, and 190.24 per 1000 person-years respectively.

**Table 2:** Incidence rate of incident NAFLD.

GGT/GDL-c	Participants (n)	NAFLD events (n)	Incidence rate (95% CI) (%)	Per 1000 person-yr
Total	11,692	1926	16.47 (15.81–17.15)	67.37
TG < 1.7mmol/L				
Q1 (<10.68)	2840	85	2.99 (2.41–3.67)	12.82
Q2 (10.68–15.39)	2641	222	8.41 (7.39–9.51)	34.36
Q3 (15.39–23.36)	2295	363	15.82 (14.37–17.35)	64.08
Q4 (≥23.36)	1628	416	25.55 (23.48–27.71)	101.95
*P* for trend				<.0001
TG ≥ 1.7 mmol/L				
Q1 (<10.68)	81	8	9.88 (4.69–17.89)	37.11
Q2 (10.68–15.39)	267	47	17.60 (13.38–22.52)	66.63
Q3 (15.39–23.36)	644	185	28.73 (25.33–32.31)	110.36
Q4 (≥23.36)	1296	600	46.30 (43.59–49.02)	190.24
*P* for trend				<.0001

NAFLD = nonalcoholic fatty liver disease, TG = triglyceride.

**Figure 2.**
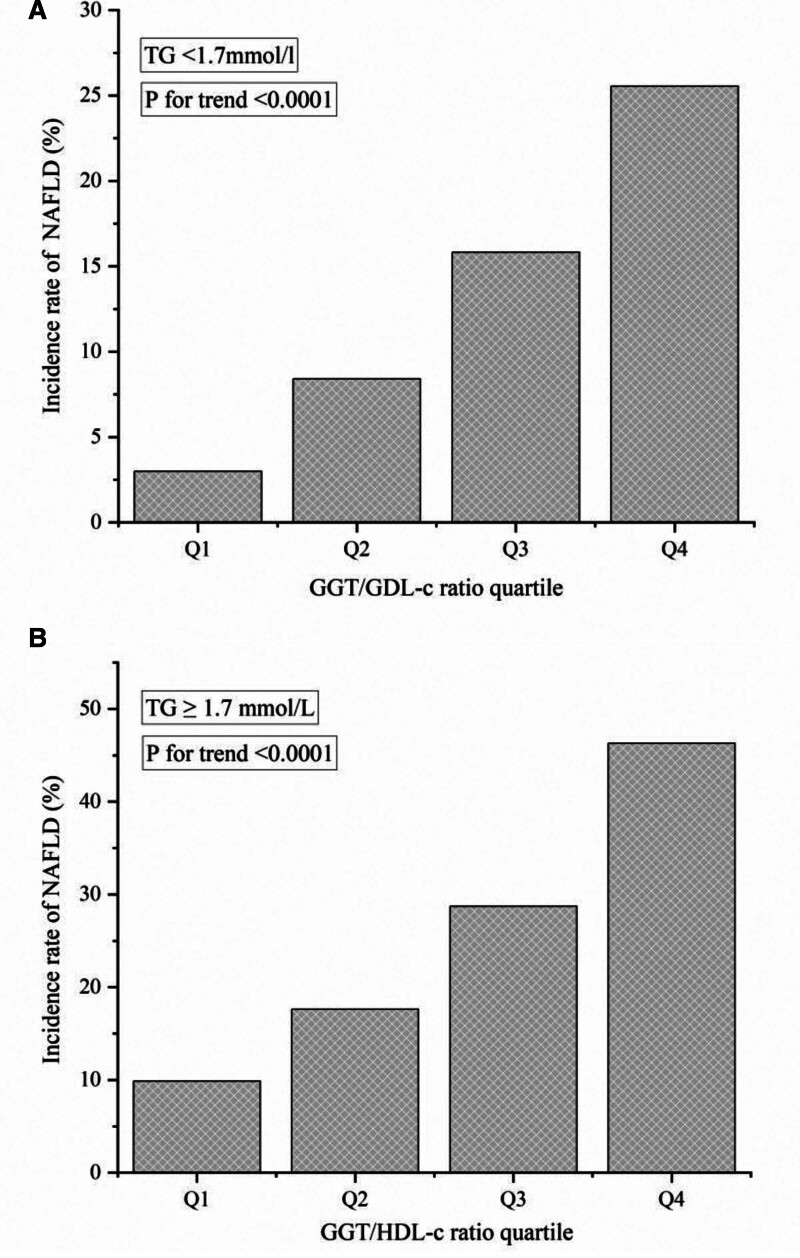
Incidence of NAFLD according to the quartiles of GGT/HDL-c in participants with non-hypertriglyceridemia (A) and with hypertriglyceridemia (B). GGT/HDL-c ratio = gamma-glutamyl transferase to high-density lipoprotein cholesterol ratio, NAFLD = nonalcoholic fatty liver disease.

In Figure 2, we found that the incidence of NAFLD was higher among participants with higher GGT/HDL-c ratios, whether in the group with TG < 1.7 mmol/l or TG ≥ 1.7 mmol/l (*P* for trend < .0001). And we also found that In 4 subgroups with different GGT/HDL-c ratios, the incidence of NAFLD in high triglyceride group was higher than that in non-high triglyceride group.

### 3.3. Association between TG and GGT/HDL-c ratio

**Figure 3.**
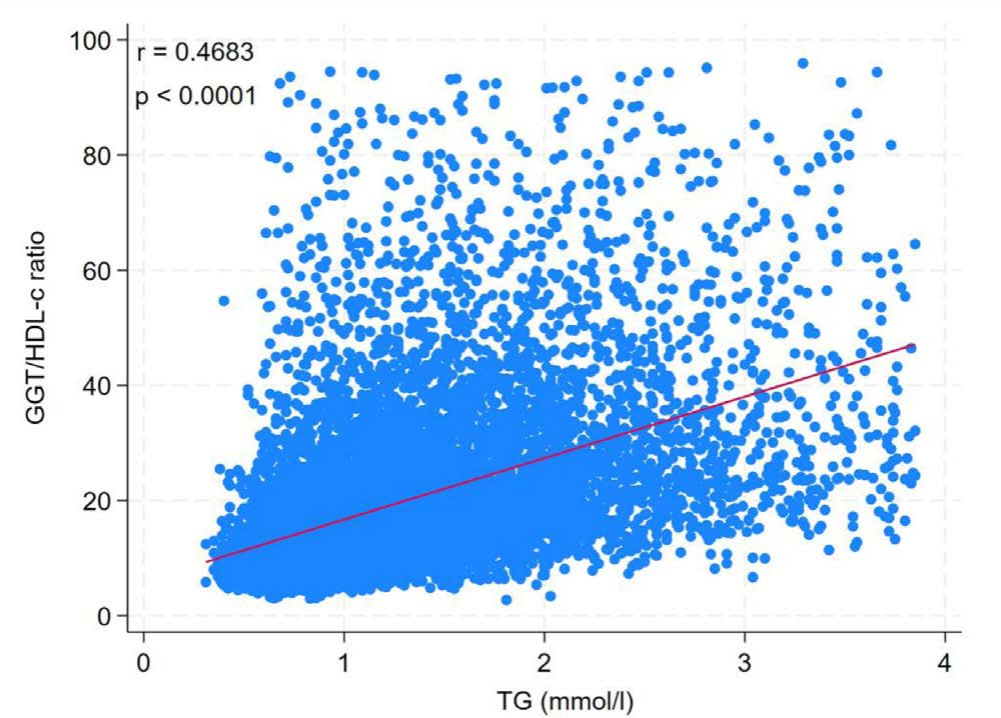
Correlation analysis of TG and GGT/HDL-c. GGT/HDL-c ratio = gamma-glutamyl transferase to high-density lipoprotein cholesterol ratio, TG = triglyceride.

Linear regression model was used to test the correlation between TG and GGT/HDL-c ratio in all participants (Table 3). In the complete model, there was a positive correlation between TG and GGT/HDL-c ratios. With an increase in TG of 1 mmol/L, GGT/HDL-c ratio had an increase of 7.36 (β = 7.36, 95% CI = 6.99–7.73, *P* < .0001). When TG is regarded as a classification variable, compared with participants with TG < 1.7 mmol/L, GGT/HDL-c ratio had an increase of 8.74 (β = 8.74, 95% CI = 8.19–9.27, *P* < .0001) in participants with TG ≥ 1.7 mmol/L. Simultaneously, there was a positive correlation between TG and the GGT/HDL-c ratio by correlation analysis (*R* = 0.4683, *P* < .0001) (Fig. 3).

**Table 3:** Association between TG and GGT/HDL-c ratio in the entire cohort.

TG (mmol/L)	Crude model	ModelⅠ	ModelⅡ
	(β, 95% CI, *P*)	(β, 95% CI, *P*)	(β, 95% CI, *P*)
TG	10.68 (10.31, 11.04) < .0001	9.03 (8.65, 9.41) < .0001	7.36 (6.99, 7.73) < .0001
TG group			
<1.7	Ref	Ref	Ref
≥1.7	13.45 (12.86, 14.04) < .0001	10.99 (10.40, 11.58) < .0001	8.74 (8.19, 9.27) < .0001
TG group			
≥1.7	Ref	Ref	Ref
<1.7	−13.45 (−14.04, −12.86) < .0001	−10.99 (−11.58, −10.40) < .0001	−8.74 (−9.29, −8.19) < .0001

Crude model adjust for: None.

Model I adjust for: sex, age, SBP, DBP, BMI.

Model II adjust for: sex, age, SBP, DBP, BMI, ALT, AST, ALB, GLB, AL, TBIL, UA, FPG, Scr, LDL-c.

ALB = albumin, ALP = alkaline phosphatase, ALT = alanine aminotransferase, AST = aspartate aminotransferase, BMI = body mass index, DBP = diastolic blood pressure, FPG = fasting plasma glucose, GGT/HDL-c ratio = gamma-glutamyl transferase to high-density lipoprotein cholesterol ratio, GLB = globulin, LDL-C = low-density lipid cholesterol, NAFLD = Nonalcoholic fatty liver disease, SBP = systolic blood pressure, Scr = serum creatinine, TBIL = total bilirubin, TG = triglyceride, UA = uric acid.

### 3.4. The association of GGT/HDL-c ratio and TG with incident NAFLD

The relationship between the GGT/HDL-c ratio, TG levels, and the risk of NAFLD was examined using COX regression models.

In the unadjusted (Crude) model, a 1-unit increase in the GGT/HDL-c ratio was associated with a 3% higher risk of NAFLD (HR = 1.030, 95% CI = 1.028–1.032, *P* < .0001). After multivariate adjustments (Model IIa), each 1-unit rise in the GGT/HDL-c ratio corresponded to a 1.3% increase in NAFLD risk (HR = 1.013, 95% CI = 1.010–1.016, *P* < .0001). When the GGT/HDL-c ratio was analyzed as a categorical variable, a strong positive correlation with NAFLD risk persisted. In Model IIa, participants in the highest quartile (Q4) had a 3.758-fold higher risk of NAFLD compared to those in the lowest quartile (Q1) (HR = 3.758, 95% CI = 2.987–4.729, *P* < .0001), with a significant trend (P for trend < 0.0001) (Table 4).

**Table 4:** Association between GGT/HGL-c and TG with risk of incident NAFLD.

	(HR, 95% CI, *P*)	(HR, 95% CI, *P*)	(HR, 95% CI, *P*)
GGT/HDL-c	Crude model	Model I	Model IIa
GGT/HDL-c ratio	1.030 (1.028–1.032) < .0001	1.020 (1.018–1.023) < .0001	1.013 (1.010–1.016) < .0001
GGT/HDL-c quartile			
Q1 (<10.68)	Ref	Ref	Ref
Q2 (10.68–15.39)	2.620 (2.069–3.317) < .0001	2.031 (1.589–2.551) < .0001	1.967 (1.550–2.496) < .0001
Q3 (15.39–23.36)	5.108 (4.099–6.366) < .0001	3.192 (2.556–3.985) < .0001	2.832 (2.256–3.556) < .0001
Q4 (≥23.36)	9.549 (7.719–11.813) < .0001	4.861 (3.914–6.036) < .0001	3.758 (2.987–4.729) < .0001
*P* for trend	<.0001	<.0001	<.0001

TG, mmol/L	Crude model	Model I	Model IIb
TG	2.197 (2.084–2.317) < .0001	1.709 (1.613–1.810) < .0001	1.537 (1.440–1.641) < .0001
TG group			
<1.7	Ref	Ref	Ref
≥1.7	2.952 (2.697–3.231) < .0001	1.974 (1.800–2.164) < .0001	1.666 (1.508–1.841) < .0001
TG group			
≥1.7	Ref	Ref	Ref
<1.7	0.339 (0.309–0.371) < .0001	0.507 (0.462–0.556) < .0001	0.600 (0.543–0.663) < .0001

Crude model adjust for: none.

Model I adjust for: sex, age, SBP, DBP, BMI.

Model IIa adjust for: sex, age, SBP, DBP, BMI, ALT, AST, ALB, GLB, ALP, TBIL, UA, FPG, Scr, LDL-c, TG.

Model IIb adjust for: sex, age, SBP, DBP, BMI, ALT, AST, ALB, GLB, ALP, TBIL, UA, FPG, Scr, LDL-c, GGT/HDL-c ratio.

ALB = albumin, ALP = alkaline phosphatase, ALT = alanine aminotransferase, AST = aspartate aminotransferase, BMI = body mass index, DBP = diastolic blood pressure, FPG = fasting plasma glucose, GGT/HDL-c ratio = gamma-glutamyl transferase to high-density lipoprotein cholesterol ratio, GLB = globulin, LDL-C = low-density lipid cholesterol, NAFLD = Nonalcoholic fatty liver disease, SBP = systolic blood pressure, Scr = serum creatinine, TBIL = total bilirubin, TG = triglyceride, UA = uric acid.

**Figure 4.**
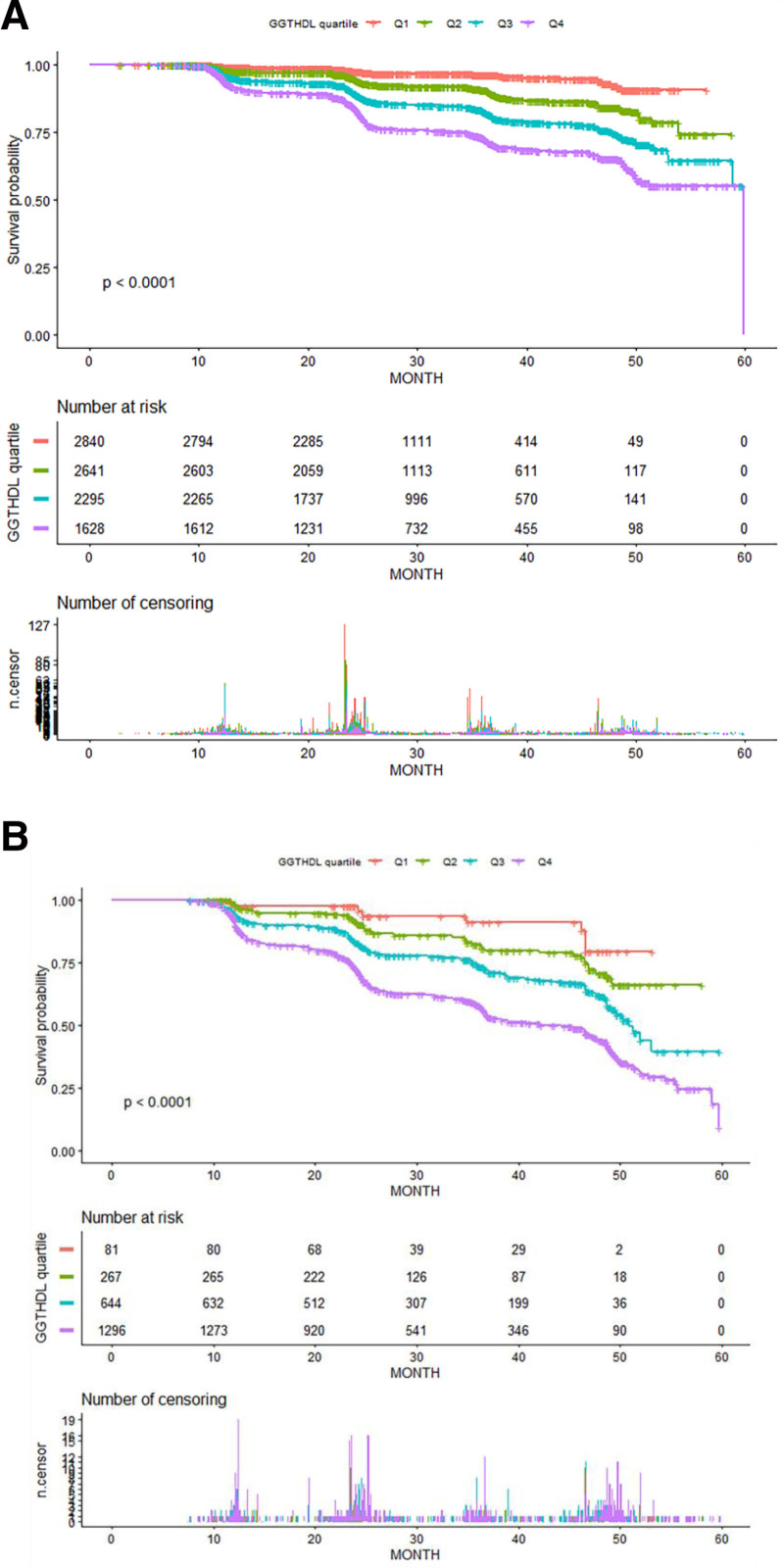
(A and B) Kaplan–Meier event-free survival curve in participants with non-hypertriglyceridemia (A) and with hypertriglyceridemia (B).

In the Crude model, a 1 mmol/L increase in TG levels was associated with a 119.7% higher risk of NAFLD (HR = 2.197, 95% CI = 2.084–2.317, *P* < .0001). After adjusting for confounders (Model IIb), each 1 mmol/L rise in TG levels was linked to a 53.7% increase in NAFLD risk (HR = 1.537, 95% CI = 1.440–1.641, *P* < .0001). When TG was categorized, participants with TG ≥ 1.7 mmol/L had a 195.2% higher risk of NAFLD in the Crude model (HR = 2.952, 95% CI = 2.697–3.231, *P* < .0001) and a 66.6% higher risk in Model IIb (HR = 1.666, 95% CI = 1.508–1.841, *P* < .0001) compared to those with TG < 1.7 mmol/L (Fig. 4).

Kaplan–Meier survival curves were used to assess NAFLD-free survival probabilities across GGT/HDL-c subgroups within each TG group (Fig. 4A and B). Results showed that NAFLD-free survival decreased significantly with higher GGT/HDL-c ratios, regardless of TG levels (log-rank test, *P* < .0001). Additionally, participants with TG < 1.7 mmol/L consistently exhibited higher NAFLD-free survival probabilities compared to those with TG ≥ 1.7 mmol/L across all GGT/HDL-c subgroups (Fig. 4).

### 3.5. The modification effect of TG on the relationship between GGT/HDL-c and the risk of NAFLD

**Figure 5.**
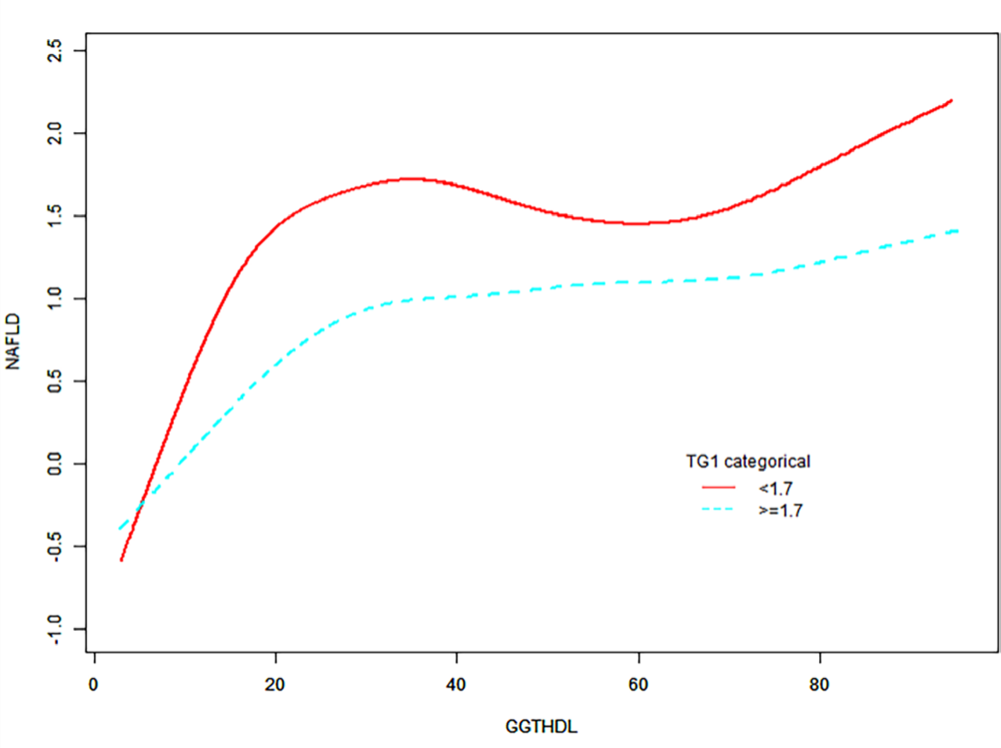
The modification effect of TG on the relationship between GGT/HDL-c and the risk of NAFLD. GGT/HDL-c ratio = gamma-glutamyl transferase to high-density lipoprotein cholesterol ratio, NAFLD = nonalcoholic fatty liver disease, TG = triglyceride.

We explored the association between the GGT/HDL-c ratio and the incidence of NAFLD using amultivariate Cox proportional hazards regression model in participants with and without hypertriglyceridemia (Table 5). In Model II, it was observed that among participants devoid of hypertriglyceridemia, the GGT/HDL-c ratio exhibited a stronger link with NAFLD incidence (HR = 1.019, 95% CI = 1.015–1.023, *P* < .0001). Conversely, this association was comparatively weaker in those with hypertriglyceridemia (HR = 1.012, 95% CI = 1.008–1.016, *P* < .0001). When we categorized the GGT/HDL-c ratio into quartiles, the results from the multivariate Cox proportional hazards regression model were consistent with those observed when treating the GGT/HDL-c ratio as a continuous variable. Based on these findings, we considered that triglycerides can influence the relationship between the GGT/HDL-c and the risk of NAFLD. The smooth curve fitting stratified by TG levels also showed the same findings (Fig. 5).

**Table 5:** Effect modification of TG on the association between GGT/HDL-c and NAFLD risk.

Incident NAFLD	Crude model	Model I	Model II
	(HR, 95% CI, *P*)	(HR, 95% CI, *P*)	(HR, 95% CI, *P*)
**GGT/HDL-c ratio**			
*TG group*			
<1.7 mmol/L	1.030 (1.026–1.033) < .0001	1.020 (1.016–1.023) < .0001	1.019 (1.015–1.023) < .0001
≥ 1.7 mmol/L	1.017 (1.013–1.020) < .0001	1.013 (1.010, 1.017) < 0.0001	1.012 (1.008–1.016) < .0001
*P* value for interaction	<.0001	.002	.011
**GGT/HDL-c quartile**			
*TG group*			
TG < 1.7 mmol/L			
GGT/HDL-c Quartile			
Q1 (<10.68)	Ref	Ref	Ref
Q2 (10.68–15.39)	2.542 (1.979–3.264) < .0001	1.961 (1.526–2.521) < .0001	2.078 (1.613–2.676) < 0.0001
Q3 (15.39–23.36)	4.724 (3.729–5.985) < .0001	2.921 (2.300–3.710) < .0001	3.096 (2.424–3.954) < 0.0001
Q4 (≥23.36)	7.355 (5.821–9.294) < .0001	3.836 (3.021–4.869) < .0001	4.061 (3.164–5.211) < 0.0001
TG ≥ 1.7 mmol/L			
GGT/HDL-c Quartile			
Q1 (<10.68)	Ref	Ref	Ref
Q2 (10.68–15.39)	1.743 (0.825–3.685) .146	1.442 (0.681–3.052) .339	1.490 (0.703–3.159) .298
Q3 (15.39–23.36)	2.888 (1.423–5.861) .003	2.269 (1.116–4.612) .024	2.277 (1.118–4.638) .023
Q4 (≥23.36)	4.970 (2.474–9.986) < .0001	3.455 (1.715–6.961) .001	3.267 (1.615–6.612) .001
*P* value for interaction	.038	.058	.034

Crude Model adjust for: None.

Model I adjust for: sex, age, SBP, DBP, BMI.

Model II adjust for: sex, age, SBP, DBP, BMI, ALT, AST, ALB, GLB, ALP, TBIL, UA, FPG, Scr, LDL-c.

ALB = albumin, ALP = alkaline phosphatase, ALT = alanine aminotransferase, AST = aspartate aminotransferase, BMI = body mass index, DBP = diastolic blood pressure, FPG = fasting plasma glucose, GGT/HDL-c ratio = gamma-glutamyl transferase to high-density lipoprotein cholesterol ratio, GLB = globulin, LDL-C = low-density lipid cholesterol, NAFLD = nonalcoholic fatty liver disease, SBP = systolic blood pressure, Scr = serum creatinine, TBIL = total bilirubin, TG = triglyceride, UA = uric acid.

### 3.6. The interactive effect of GGT/HDL-c and TG on the risk of NAFLD

To explore the potential interaction between the GGT/HDL-c ratio and triglycerides (TG) on the risk of NAFLD, participants were divided into 8 groups based on GGT/HDL-c quartiles and the presence of hypertriglyceridemia. The groups included: those with a GGT/HDL-c ratio < 10.68 (Q1) without hypertriglyceridemia, those with a ratio ≥ 10.68 and <15.39 (Q2) without hypertriglyceridemia, those with a ratio ≥ 15.39 and <23.36 (Q3) without hypertriglyceridemia, those with a ratio ≥ 23.36 (Q4) without hypertriglyceridemia, and corresponding groups with hypertriglyceridemia (Table 6).

**Table 6:** Interaction of TG and GGT/HDL-c and their association with NAFLD.

Exposure	Crude model	Model I	Model II
	(HR, 95% CI, *P*)	(HR, 95% CI, *P*)	(HR, 95% CI, *P*)
Interaction of TG and GGT/HDL-c			
TG < 1.7 mmol/L and Q1 (<10.68)	Ref	Ref	Ref
TG < 1.7 mmol/L and Q2 (10.68–15.39)	2.537 (1.976, 3.258) < 0.0001	2.012 (1.566, 2.585) < 0.0001	2.101 (1.633, 2.703) < 0.0001
TG < 1.7 mmol/L and Q3 (15.39–23.36)	4.704 (3.714, 5.959) < 0.0001	3.059 (2.411, 3.881) < 0.0001	3.176 (2.495, 4.044) < 0.0001
TG < 1.7 mmol/L and Q4 (≥23.36)	7.333 (5.805, 9.264) < 0.0001	4.084 (3.222, 5.175) < 0.0001	4.161 (3.261, 5.310) < 0.0001
TG ≥ 1.7 mmol/L and Q1 (<10.68)	2.727 (1.321, 5.630) 0.007	2.087 (1.010, 4.311) 0.047	2.081 (1.006, 4.304) 0.048
TG ≥ 1.7 mmol/L and Q2 (10.68–15.39)	4.791 (3.362, 6.827) < 0.0001	2.811 (1.969, 4.014) < 0.0001	2.977 (2.079, 4.262) < 0.0001
TG ≥ 1.7 mmol/L and Q3 (15.39–23.36)	7.928 (6.129, 10.255) < 0.0001	4.373 (3.371, 5.673) < 0.0001	4.588 (3.517, 5.983) < 0.0001
TG ≥ 1.7 mmol/L and Q4 (≥23.36)	13.715 (10.923, 17.220) < 0.0001	6.380 (5.057, 8.049) < 0.0001	6.662 (5.237, 8.474) < 0.0001

Crude Model adjust for: None.

Model I adjust for: sex, age, SBP, DBP, BMI.

Model II adjust for: sex, age, SBP, DBP, BMI, ALT, AST, ALB, GLB, ALP, TBIL, UA, FPG, Scr, LDL-c.

ALB = albumin, ALP = alkaline phosphatase, ALT = alanine aminotransferase, AST = aspartate aminotransferase, BMI = body mass index, DBP = diastolic blood pressure, FPG = fasting plasma glucose, GGT/HDL-c ratio = gamma-glutamyl transferase to high-density lipoprotein cholesterol ratio, GLB = globulin, LDL-C = low-density lipid cholesterol, NAFLD = Nonalcoholic fatty liver disease, SBP = systolic blood pressure, Scr = serum creatinine, TBIL = total bilirubin, TG = triglyceride, UA = uric acid.

**Figure 6.**
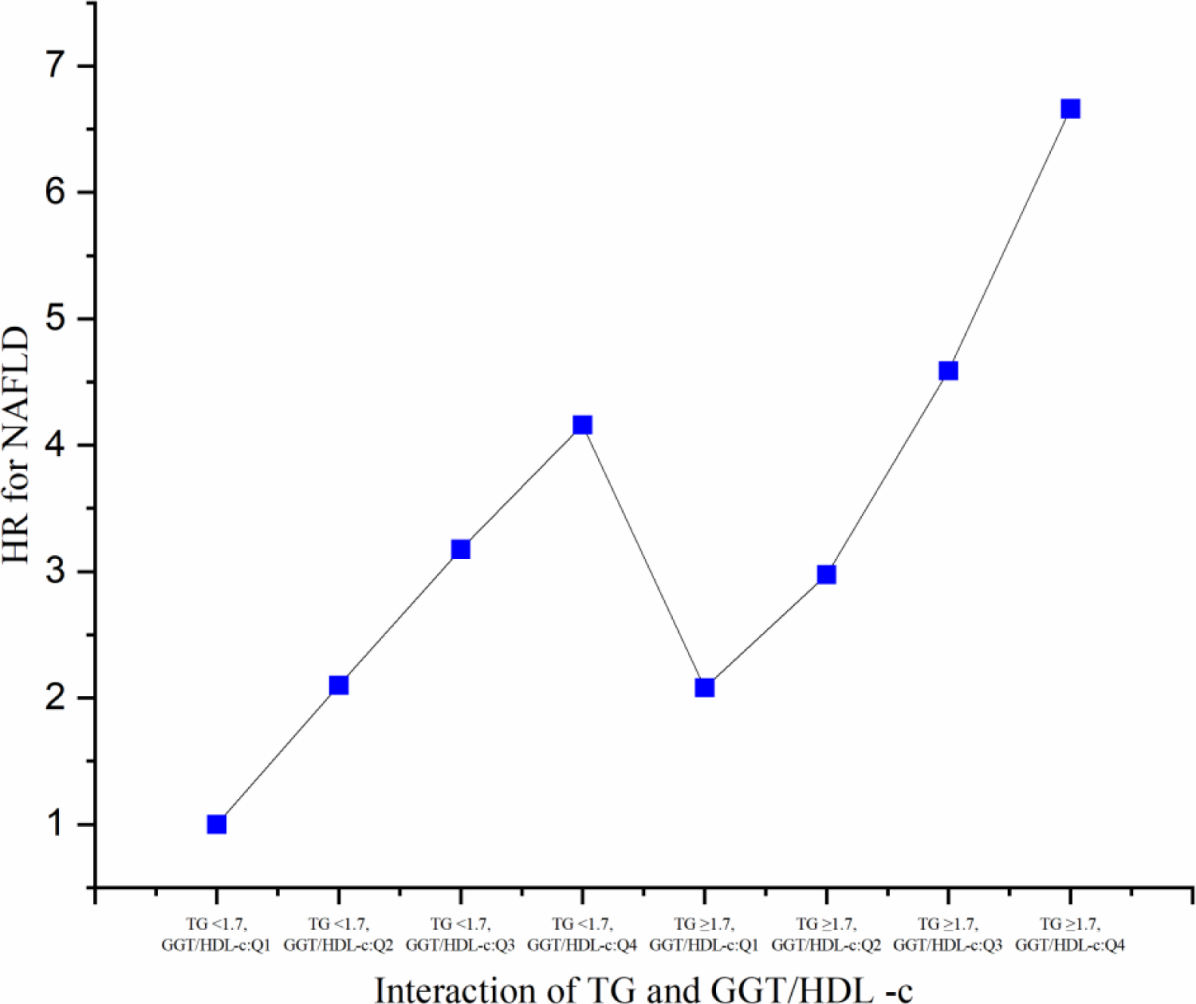
The interactive effect of GGT/HDL-c and TG on the risk of NAFLD. GGT/HDL-c ratio = gamma-glutamyl transferase to high-density lipoprotein cholesterol ratio, NAFLD = nonalcoholic fatty liver disease, TG = triglyceride.

A multivariate Cox proportional hazards regression model revealed significant associations between these groups and NAFLD risk. Compared to non-hypertriglyceridemic individuals with a GGT/HDL-c ratio < 10.68 (Q1), those with elevated GGT/HDL-c ratios and hypertriglyceridemia showed a markedly higher risk of NAFLD. Specifically, hypertriglyceridemic participants with a GGT/HDL-c ratio ≥ 23.36 (Q4) had the greatest risk (HR = 6.662, 95% CI = 5.237–8.474, *P* < .0001). Hypertriglyceridemic individuals with the same GGT/HDL-c ratio exhibited a significantly elevated risk compared to their non-hypertriglyceridemic counterparts. These findings indicate that the combined effect of GGT/HDL-c and TG levels may play a critical role in influencing NAFLD risk (Fig. 6).

### 3.7. The results of subgroup analyses

For participants exhibiting TG levels ≥ 1.7 mmol/L, subgroup analysis did not reveal any notable interactions across categories of age, gender, FPG, systolic, or DBP. Conversely, among those with TG levels < 1.7 mmol/L, significant interactions were detected in relation to BMI and FPG. A heightened association between the GGT/HDL-c ratio and NAFLD incidence was observed in individuals with a BMI of <24 kg/m² and an FPG of 6.1 mmol/L or lower (Table 7).

**Table 7:** subgroup analysis and interaction analysis.

Subgroup	TG < 1.7 mmol/L (HR, 95% CI)	*P* for interaction	TG ≥ 1.7 mmol/L (HR, 95% CI)	*P* for interaction
Age, yr				
<30	1.021 (1.010–1.031)	.9421	1.011 (1.000–1.023)	.6162
30 to 40	1.015 (1.006–1.023)		1.017 (1.010–1.025)	
40 to 50	1.017 (1.009–1.026)		1.012 (1.003–1.021)	
50 to 60	1.020 (1.010–1.031)		1.007 (0.995–1.019)	
60 to 70	1.017 (0.997–1.037)		1.004 (0.990–1.017)	
≥70	1.016 (1.003–1.029)		1.003 (0.988–1.018)	
Sex, yr				
Male	1.016 (1.011–1.022)	.6363	1.013 (1.007–1.018)	.2291
Female	1.019 (1.013–1.026)		1.010 (1.004–1.016)	
BMI, kg/m^2^				
<24	1.027 (1.023–1.032)	<.0001	1.012 (1.007–1.017)	.1786
≥24	1.010 (1.002–1.017)		1.019 (1.011–1.026)	
FPG, mmol/L				
≤6.1	1.020 (1.016–1.024)	.0312	1.012 (1.008–1.017)	.9427
>6.1	1.007 (0.996–1.019)		1.014 (1.001–1.027)	
SBP, mm Hg				
<140	1.020 (1.015–1.024)	.0874	1.011 (1.007–1.016)	.4504
≥140	1.013 (1.004–1.022)		1.017 (1.008–1.026)	
DBP, mm Hg				
<90	1.019 (1.015–1.024)	.4216	1.011 (1.007–1.016)	.1748
≥90	1.015 (1.004–1.025)		1.016 (1.005–1.026)	

Above model adjusted for sex, age, SBP, DBP, BMI, ALT, AST, ALB, GLB, ALP, TBIL, UA, FPG, Scr, LDL-c.

In each case, the model is not adjusted for the stratification variable.

ALB = albumin, ALP = alkaline phosphatase, ALT = alanine aminotransferase, AST = aspartate aminotransferase, BMI = body mass index, DBP = diastolic blood pressure, FPG = fasting plasma glucose, GLB = globulin, LDL-C = low-density lipid cholesterol, SBP = systolic blood pressure, Scr = serum creatinine, TBIL = total bilirubin, TG = triglyceride, UA = uric acid.

## 4. Discussion

The association between triglycerides (TG) and the GGT/HDL-c ratio with the risk of NAFLD in nonobese individuals was investigated in this cohort study. Our findings demonstrated that both TG levels and the GGT/HDL-c ratio were positively associated with the risk of developing NAFLD. In addition, TG levels showed a positive correlation with the GGT/HDL-c ratio. When participants were stratified by the presence of high triglycerides. In participants without high triglycerides, the association between the GGT/HDL-c ratio and NAFLD risk was stronger (HR = 1.019, 95% CI = 1.015–1.023, *P* < .0001) compared to those with high triglycerides (HR = 1.012, 95% CI = 1.008–1.016, *P* < .0001). The most important finding is that there was an interaction effect between TG and GGT/HDL-c ratio on the risk of NAFLD. Compared to participants with a GGT/HDL-c ratio < 10.68 (Q1) without high triglycerides, those with a GGT/HDL-c ratio ≥ 23.36 (Q4) and high triglycerides had a 5.662-fold increased risk of NAFLD (HR = 6.662, 95%CI = 5.237–8.474, *P* < .0001).

The potential connection between GGT/HDL-c levels and NAFLD has not been thoroughly investigated. Nonetheless, evidence from a cross-sectional Chinese study suggests a possible association worthy of further exploration,^[16]^ included 6326 participants, assigning 1813 to the NAFLD group and 4513 to the non-NAFLD group. The study findings revealed a significant association between the GGT/HDL-C ratio and the prevalence of NAFLD. Specifically, for every 1-unit increase in the GGT/HDL-C ratio, the prevalence of NAFLD increased by 0.3%. In another cross-sectional study from Lima, Peru,^[25]^ the researchers investigated 249 adults with obesity undergoing bariatric surgery. They found that the optimal cutoff value for the GGT/HDL-C ratio was 20.5 U/mmol and the prevalence of NAFLD increased by 14% among the group with an elevated GGT/HDL-C ratio. In our study, we found that the GGT/HDL-c ratio was positively associated with the incidence of NAFLD in nonobese individuals. Specifically, each additional 1 unit in the GGT/HDL-c ratio was associated with a 1.3% increased risk of NAFLD after multivariate adjustments (HR = 1.013, 95% CI = 1.010–1.016, *P* < .0001).

Numerous studies have demonstrated that different lipid parameters are linked to the onset of NAFLD.^[26–28]^ Specifically, TG and HDL-C are independently correlated with NAFLD risk. TG is positively correlated with NAFLD development, while HDL-C is negatively correlated with NAFLD development. A study^[29]^ investigating the relationship between high triglyceride waist phenotype and NAFLD found that individuals with normal waist circumference but elevated TG levels had a 96% increased prevalence of NAFLD compared to those with normal waist circumference and TG levels after adjusting for confounding factors. Our study found that high TG levels were positively correlated with the incidence of NAFLD, which is consistent with the results of previous relevant studies. Moreover, GGT plays a significant role in determining NAFLD risk, with adults having higher GGT levels being more prone to developing the condition.^[30]^ In addition, GGT levels are linked to liver fibrosis in NAFLD patients.^[31]^

The current evidence on the impact of TG on the relationship between GGT/HDL-c and the risk of NAFLD is still limited. Our study analyzed the effect of TG on the relationship between GGT/HDL-c and the risk of NAFLD, while a relatively weaker association was found in the hypertriglyceridemia group (HR = 1.012, 95% CI = 1.008–1.016, *P* < .0001). The smoothing curve fitting results also revealed the same finding. Our results suggest that TG can affect the relationship between GGT/HDL-c and the risk of NAFLD. The modulating effect of TG on the relationship between GGT/HDL-c and NAFLD has important clinical implications, indicating that reducing the GGT/HDL-c ratio in nonobese individuals may help lower the incidence of NAFLD. For patients without hypertriglyceridemia, the reduction in NAFLD risk is more pronounced by lowering the GGT/HDL-c ratio. Additionally, the interaction between TG and GGT/HDL-c can influence the risk of NAFLD, with a significantly elevated NAFLD risk observed in participants with both a high GGT/HDL-c ratio and high triglyceride levels.

The exact mechanism by which TG influence the correlation between GGT/HDL-c and nonalcoholic fatty liver disease risk is not yet fully understood. Elevated triglyceride levels can directly influence lipid accumulation in the liver.^[32]^ Research indicates that in NAFLD patients, the liver and plasma lipid and fatty acid sources originate from peripheral fatty acids and new lipid metabolism pathways.^[33]^ Additionally, hypertriglyceridemia can lead to an increase in TG-enriched HDL levels, which in turn can cause HDL-c dysfunction and promote the development of NAFLD.^[34]^ Research has indicated^[35]^ that in metabolically-associated NAFLD patients, HDL-c may exert a protective effect by facilitating the clearance of unhealthy lipids in the liver. However, when HDL-c levels are abnormal, it may be unable to effectively clear these unhealthy lipids, resulting in heightened lipid buildup in the liver and thus fostering the progression of NAFLD.^[36]^ Furthermore, NAFLD is closely related to insulin resistance,^[32,37]^ which is also an important characteristic of hypertriglyceridemia.^[38]^ Insulin resistance can intensify lipid accumulation in the liver and potentially advance NAFLD by boosting cholesterol metabolism in the liver.^[39]^ GGT is a common liver function marker, often used to assess liver injury. Studies have shown that GGT is a sensitive marker of insulin resistance in adults, and serum GGT levels are an independent predictor of HOMA-IR apart from fatty degeneration.^[40]^ Epidemiological data have also shown that elevated GGT is associated with the incidence of metabolic syndrome, and this association is attenuated by the severity of insulin resistance.^[41]^ A cross-sectional study from China also observed a significant positive correlation between GGT and HOMA-IR.^[42]^ Therefore, we propose that the association between triglyceride levels, the GGT/HDL-c ratio, and the risk of NAFLD may be linked to enhanced hepatic fat accumulation and the development of insulin resistance.

While our findings elucidate the TG–GGT/HDL-c–NAFLD interplay, cautious extrapolation is warranted given potential population-specific mechanisms. Studies indicate ethnic disparities in NAFLD pathogenesis – for instance, African Americans exhibit distinct hepatic insulin resistance patterns compared to East Asians at comparable BMI levels, likely influenced by genetic variants such as PNPLA3 and TM6SF2 with population-specific effects. Furthermore, lifestyle factors like dietary composition may modulate these associations: Western populations with higher saturated fat intake demonstrate stronger diet-NAFLD linkages than Asian cohorts, suggesting our findings in carbohydrate-predominant diets may not fully capture lipid synergy in high-fat contexts.^[43]^ Geographic heterogeneity in comorbidities (e.g., prevalent early-onset diabetes in Middle Eastern groups) could further alter risk gradients. Future studies should prioritize multi-ethnic cohorts to validate these interactions across genetic and environmental contexts, mechanistic models comparing hepatic lipid flux under varying TG/GGT/HDL-c thresholds, and culturally adapted interventions targeting high-risk subgroups defined by population-specific cutoffs.

### 4.1. Study strengths and limitations

This study possesses several notable strengths. First, it was based on a large-scale cohort of nonobese Chinese adults, which enhances the credibility and generalizability of the findings within this population. Second, by stratifying participants according to TG levels, we were able to identify that TG modifies the association between the GGT/HDL-c and the risk of nonalcoholic fatty liver disease (NAFLD). Third, to our knowledge, this is the first investigation to demonstrate a significant interaction between TG and GGT/HDL-c in relation to NAFLD risk, providing valuable insights that may inform clinical strategies for NAFLD prevention. In addition, we employed multiple imputation methods to address missing data, which helped to maximize statistical power and minimize potential bias caused by incomplete covariate information.

However, certain limitations should be acknowledged. As an observational study, our research cannot confirm causality between the variables examined. Despite adjusting for numerous known confounders – including sex, age, systolic and DBP, BMI, alanine aminotransferase, aspartate aminotransferase, albumin, globulin, alkaline phosphatase, total bilirubin, UA, FPG, serum creatinine, and low-density lipoprotein cholesterol – residual confounding from unmeasured factors remains possible. Furthermore, NAFLD diagnosis in this study was based on abdominal ultrasound rather than liver biopsy, which may have led to underdiagnosis of mild hepatic steatosis. Finally, because the study population was limited to nonobese Chinese adults, caution should be exercised when generalizing these findings to other ethnic groups or individuals with a BMI > 25 kg/m².

## 5. Conclusion

The findings of this study indicate that TG levels play a modifying role in the relationship between the GGT/HDL-c and the risk of NAFLD among nonobese Chinese individuals. Notably, in participants with normal TG concentrations, the association between GGT/HDL-c and NAFLD risk was more pronounced. Furthermore, an interaction effect was observed between GGT/HDL-c and TG levels, where individuals exhibiting both elevated GGT/HDL-c ratios and hypertriglyceridemia demonstrated a markedly increased risk of developing NAFLD. For this population, we recommend that lifestyle interventions - including a Mediterranean diet pattern that emphasizes anti-inflammatory foods and an aerobic resistance exercise regimen – prioritize the simultaneous regulation of lipids and liver markers. For high-risk subgroups with co-existing lipid abnormalities, adjuvant medication (e.g., omega-3 fatty acids, liver protectants) may extend the risk reduction. These discoveries bridge an important knowledge gap in nonobese NAFLD etiology and provide a biomarker-driven framework for early prevention. Future studies should validate these thresholds in multi-ethnic cohorts and explore targeted therapies to disrupt the TG–GGT/HDL-c synergy.

## Acknowledgments

The data and methodological description for this secondary analysis are mainly derived from the following study: Q. Sun, S. J. Wu, W. Y. Liu, L. R. Wang, Y. R. Chen, D. C. Zhang, M. Braddock, K. Q. Shi, D. Song, and M. H. Zheng, Association of low-density lipoprotein cholesterol within the normal range and NAFLD in the nonobese Chinese population: a cross-sectional and longitudinal study, BMJ Open, 6 (12), e013781. doi: 10.1136/bmjopen-2016-013781 (28). We express our heartfelt thanks to the authors of this study.

## Author contributions

**Conceptualization:** Aihua Chen, Cishuang Fu, Haiying Chen.

**Data curation:** Aihua Chen, Haiying Chen.

**Formal analysis:** Haiying Chen.

**Funding acquisition:** Cishuang Fu, Haiying Chen.

**Investigation:** Aihua Chen, Cishuang Fu, Haiying Chen.

**Methodology:** Aihua Chen, Cishuang Fu, Haiying Chen.

**Supervision:** Aihua Chen, Cishuang Fu.

**Validation:** Cishuang Fu.

**Visualization:** Cishuang Fu.

**Writing – original draft:** Aihua Chen, Haiying Chen.

**Writing – review & editing:** Aihua Chen, Haiying Chen.
